# Integrated Oxidized-Hyaluronic Acid/Collagen Hydrogel with β-TCP Using Proanthocyanidins as a Crosslinker for Drug Delivery

**DOI:** 10.3390/pharmaceutics10020037

**Published:** 2018-03-21

**Authors:** Yang Wei, Yu-Han Chang, Chung-Jui Liu, Ren-Jei Chung

**Affiliations:** 1 Department of Chemical Engineering and Biotechnology, National Taipei University of Technology (Taipei Tech), Taipei 106, Taiwan; wei2@g.clemson.edu (Y.W.); jackjefferyjack@gmail.com (C.-J.L.); 2 Bone and Joint Research Center, Chang Gung Memorial Hospital, Linko 244, Taiwan; yuhanchang2012@gmail.com; 3 Department of Orthopaedic Surgery, Chang Gung Memorial Hospital, Linko 244, Taiwan; 4 College of Medicine, Chang Gung University, Taoyuan 333, Taiwan

**Keywords:** composite hydrogel, drug delivery GBR, nature cross-linking agent

## Abstract

The susceptibility of guided bone regeneration (GBR) material to infection by pathogens at wound sites during bone healing has often been overlooked. The objective of this study was the synthesis and characterization of a potential material for antibacterial GBR application. In the current study, the mechanical strength and biocompatibility of a composite restoration material—made of oxidized hyaluronic acid (HA)/type I collagen hydrogel integrated with tricalcium phosphate (β-TCP) using a natural crosslinking agent, oligomeric proanthocyanidins (OPCs)—were evaluated. The suitability of the material as a carrier matrix for antibacterial applications was evaluated by following the drug-release profile of tetracycline loaded within the composite. Results indicated that this composite material had a high swelling ratio of 420% and mechanical strength of 25 kPa while remaining at more than 60% of the weight after 30 days of an in vitro degradation test with good biocompatibility in promoting the proliferation of MG-63 cells. Drug release studies further showed that 93% of the tetracycline was released after 5 days, which supports this GBR material’s capability to release antibacterial drugs while keeping other required GBR material design functions.

## 1. Introduction

Chronic periodontitis is a serious gum infection that may trigger a chronic inflammatory disorder that promotes bone degradation, weakening the alveolar bone surrounding the teeth, potentially leading to tooth loss [1,2]. The goal of periodontitis treatment is to thoroughly clean the pockets around teeth and prevent damage to the surrounding bone [2,3,4]. More advanced periodontitis treatment may require invasive procedures such as guided bone regeneration (GBR), which allows for the regrowth of bone that was destroyed by bacteria [5]. GBR procedures often involve the insertion of a material between the soft tissue and the bone defect in order to prevent unwanted tissue from entering the healing area, allowing the bone to grow back and for the recruitment of osteoprogenitor cells and osteoblasts to migrate to the surrounding bone [6,7]. The material serves as a scaffold for cell migration to control the release of drugs or growth factor and guide the bone formation into a desired shape [8]. Although GBR has been a very effective oral therapeutic method to regenerate bone tissue, a couple of limitations exist in the GBR material design that remain to be solved.

Biopolymers such as bioresorptive collagen were commonly used in GBR to avoid the need for a revision surgery to remove the implant [9]. However, collagen lacks the mechanical strength and is prone to enzymatic degradation from macrophages, polymorph nuclear leukocytes, and bacteria [10,11]. Various synthetic cross-linkers such as glutaraldehyde and epoxy compounds have been used to increase its tensile strength and enzymatic degradation but with concerns regarding high cytotoxicity [12,13]. Therefore, the oxidation of another natural polysaccharide, hyaluronic acid (HA), a major component of the extracellular matrix with the critical function of cell migration, was used as a macromolecular crosslinker to enhance the strength of collagen hydrogel in this study [14].

On the other hand, ceramic materials such as tricalcium phosphate (TCP) or hydroxyapatite have also been used in GBR to regenerate bone tissue because of their superior mechanical properties, tissue compatibility and osteoconductive properties but with the limitation of lacking any osteoinductive or osteogenic abilities when used alone [8,15].

Therefore, to synergize both materials for an integrated GBR, a composite material might be a better solution in terms of its biological compatibility and mechanical property. For example, various combinations of inorganic/organic hybrid materials such as TiO_2_/polycaprolactone (PCL), zirconia/PCL, silica/chlorogenic acid, ZrO_2_/PEG and SiO_2_/PCL hybrid materials have been proposed [16]. These combinations are proposed as biomaterials with different properties of polymeric materials introduced into inorganic networks to improve their biological and mechanical features, supported by either experimental or theoretical data [16].

In the case of the integrated GBR composite material, collagen with tricalcium phosphate (TCP) or hydroxyapatite has been used in GBR procedures to mimic the natural composition of bone [9], with the scaffold made of the combination of collagen and ceramics reported to promote osteogenesis in both the in vitro and in vivo biological evaluations compared to traditional GBR design [17,18]. The addition of collagen to a ceramic structure thus provides many advantages for surgical applications and the ability to favor clot formation and the stabilization of alveolar bone regeneration [8]. In addition to collagen, porous ceramic scaffolds such as TCP have also been used with hyaluronic acid (HA) for alveolar socket preservation with good success which exhibits more efficiently in osteoconduction when compared to TCP samples without HA [19,20]. Most importantly, these integrated composite scaffolds with interconnected porous structures can locally release growth factors, cells and drugs from the scaffolds for enhanced bone formation [8,18,21], and in situ cell transplantation of the central nervous system (CNS) for applications in Parkinson’s and Alzheimer’s diseases [22], which may further prevent the susceptibility of GBR material to infection by pathogens at wound sites during bone healing, which has been seldomly considered but leads to a decrease in the amount of regenerated bone especially in the case of chronic periodontitis [23].

The objective of this study was the synthesis and in vitro characterization of a composite material containing β-tricalcium phosphate (β-TCP) in an oxidized hyaluronic acid (HA)/type I collagen hydrogel using oligomeric proanthocyanidins (OPCs) as natural cross-linking agents. The material was evaluated for its mechanical strength, swelling ratio and in vitro degradation rate while its biocompatibility was evaluated using MG-63 cell lines. The utility of the matrix as a drug delivery carrier was evaluated by following the elution of tetracycline hydrochloride (TH) that was entrapped within the composite as an antibacterial GBR material for the treatment of chronic periodontitis [24,25].

## 2. Experimental Section

In this section, we present procedures to make the composite restoration material (CHTP)—integrated oxidized hyaluronic acid (HA)/type I collagen hydrogel with β-tricalcium phosphate (β-TCP)—using a natural crosslinking agent—oligomeric proanthocyanidins (OPCs). Then, we address the protocols to evaluate OPCs’ mechanical strength, swelling ratio and in vitro degradation rate as well as their biocompatibility using MG-63 cell lines and their drug-releasing capability.

### 2.1. Materials

All materials used in this study were purchased from Sigma-Aldrich Inc. (St. Louis, MO, USA) unless otherwise stated. Oligomeric proanthocyanidins (OPCs) were purchase from Compson Trading Co., Ltd. (Taichung, Taiwan). Type I collagen was purchased from Coreleader Biotech Co., Ltd. (New Taipei City, Taiwan). Hyaluronic acid (HA) was purchased from Coreleader Biotech Co., Ltd. (New Taipei City, Taiwan) with averaged molecular weight = 900 kDa. Ethylene glycol (EG) was obtained from Echo Chemical CO., Ltd. (Miaoli, Taiwan). Dimethyl sulfoxide (DMSO) was purchased from Thermo Fisher Scientific Inc. (Waltham, MA, USA). Beta-tricalcium phosphate (β-TCP) was from J.O. Corporation (New Taipei City, Taiwan).

In the following sections, different polymeric composites were prepared and evaluated separately with a quick summary of their abbreviations in Table 1.

### 2.2. Preparation and Characterizations of CH Polymers

#### 2.2.1. Oxidized HA (oxi-HA)

Hyaluronic acid (HA) was dissolved in double-distilled water (DDW) at room temperature and oxidized with sodium periodate. Briefly, 400 mL of 10 mg/mL HA solution was gently mixed with 40 mL of sodium periodate (2.67% (*w*/*v*)) and kept in the dark for 24 h. 2 mL of Ethylene glycol was then added to stop the oxidation reaction. The final oxidized HA (oxi-HA) was obtained by dialysis (MWCO = 3500) against DDW for two days and lyophilized.

#### 2.2.2. CH Polymers

Oxi-HA from the previous step was then mixed and interacted with the collagen solution (7.5 mg/mL in 0.5 M acetic acid) at different concentrations: 10%, 20%, 30%, 35% and 40% (*w*/*w*)) using a vortex mixer at 2500 rpm for 10 min to make the oxi-HA/collagen polymer (CH) solution and lyophilized for one day to have the various CH polymers prepared. For example, CH-35% presents that oxi-HA was mixed and interacted with the collagen solution at 35% (*w*/*w*).

#### 2.2.3. Pretreatment of Collagen Samples

In this study, collagen samples were pretreated with pepsin to remove possible immunogenicity due to its terminal amino acids [26]. The process was as follows: Type I collagen (7.5 mg/mL) was dissolved into 0.5 M acetic acid and mixed at 4 °C for 24 h. Pepsin (Sigma-Aldrich Inc., St. Louis, MO, USA)) was then added (the approximate ratio of enzyme to collagen was 1:400) to make the final concentration of the collagen solution 0.75 mg/mL at 4 °C for another 24 h. Powders of sodium chloride were then added at 4 °C for another 24 h and made a 2.5 M final concentration in the collagen solution from previous steps which precipitated the collagen. Following this, a Z300 centrifuge (Hermle, Wehingen, Germany) at 2500 rpm for 5 min proceeded with the precipitated pallets moving into the dialysis bag (MWCO = 13 kDa) against 0.05 M acetic acid for 3 days. Thereafter, the dialysis bag was placed into the potassium phosphate buffer (PBS; 1×, pH 7.4) for another 3 days to make the collagen with the terminal immunogenicity removed and stored at 4 °C.

#### 2.2.4. Crosslinking Index

A ninhydrin (NHN) assay was used to determine the amount of free amino groups of CH polymers. The test sample (e.g., 10 mg of CH) was weighed and heated at 90 °C with 1 mL of ninhydrin solution for 15 min. After the test solution was cooled to room temperature and diluted in 95% ethanol, the optical absorbance of the solution was recorded with an ultraviolet-visible spectrophotometer (Gene-Quant 1300, GE Healthcare Life Science, Pittsburgh, PA, USA) at 570 nm. The amount of free-NH_2_ groups in the CH before (Cb) and after (Ca) a cross-linking reaction is proportional to the optical absorbance of the solution. The degree of cross-linking of the CH polymer was calculated according to Equation (1). Results were the average of three independent measurements.

(1) Crosslinking index (%)=[(Cb−Ca)/Ca] × 100

#### 2.2.5. Preparation of Hydrogel

**CH** polymer solutions from previous steps were left steady at 4 °C for one hour and kept in a mold in a fixed shape for 24 h at −20 °C and lyophilized thereafter for another 24 h after which the sample with a fixed shape was inserted into the phosphate-buffered saline (1× PBS, pH = 7.4) at the concentration of 6% (*w*/*v*) to make the hydrogel. PBS was selected as the ideal solvent for the hydrogels under physiological conditions [27].

#### 2.2.6. Characterizations Using Fourier Transform Infrared Spectrometer (FTIR) and Scanning Electron Microscope (SEM)

A Spectrum GX FT-IR (PerkinElmer, Waltham, MA, USA) with a universal attenuated total reflectance (UATR) attachment was used to identify the functional groups of the HA, **oxi-HA**, collagen and CH polymers. A lyophilized sample from each of these polymers was placed on a zinc selenide crystal and the pressure tip was gently pressed down to keep the sample in full contact with the crystal. Spectra were obtained by recording four scans in the range 500–4000 cm^−1^ with a resolution of 8 cm^−1^.

The morphology and porous structure of the hydrogel were observed by scanning electron microscopy (SEM) (Model S-3000H, Hitachi, Tokyo, Japan). Lyophilized hydrogel was cooled in liquid nitrogen to enhance its brittleness, and then quickly fractured to expose the internal structure. Fractured samples, 5 × 5 mm^2^ in shape, were placed on double-sided tape and sputter-coated with palladium and gold to a thickness of 100 Å before the observation.

### 2.3. Preparation of Composite Materials, CHT and CHTP

CH hydrogel made of 35% (*w*/*w*) of oxi-HA in collagen solution (CH-35%) from the previous step was chosen as the raw material for further polymeric material synthesis. Firstly, CH-35% was mixed with tricalcium phosphate (β-TCP) at different weight ratios (i.e., CH-35%: β-TCP (*w*/*w*) = 0.9:0.1, 0.8:0.2, 0.7:0.3 and 0.6:0.4) using a vortex mixer for 10 min and stored at −20 °C for one day and lyophilized for 24 h to make various CHT composite polymers. For example, CHT-30% represents the weight percentages of β-TCP versus CH-35% within a CHT material, which was chosen as the raw material for further CHTP synthesis via soaking CHT-30% into a 5% (*w*/*v*) OPCs solution (i.e., OPCs dissolved in a 5% (*w*/*v*) citric acid aqueous solution) for four hours. It was then washed with DDW to remove residue OPCs then stored at −20 °C for one day and lyophilized for 24 h to make OPCs-crosslinked composites, CHTP.

### 2.4. Degradation and Swelling Properties of Composite Material

Swelling and degradation studies were conducted on composite materials in phosphate buffered saline (1× PBS, pH = 7.4) at 37 °C. Briefly, 0.01 g of lyophilized material was weighed to obtain the dry weight (W_d_) and soaked thereafter in 1.0 mL of PBS for both of the swelling and degradation tests. For the swelling test, at each specific time point, the hydrogel was removed, blotted gently with filter paper to remove surface water, and the swollen hydrogel was weighed again (W_w_), with the swelling percentage calculated using the formula: [28,29,30].

(2) (Ww−Wd)/Wd × 100%

For the degradation test, the degradation percentage was calculated using the formula:

(3) (Wd−Wf)/Wd × 100%

where W_f_ is the measured dry weight of the lyophilized hydrogel samples after soaking in 1.0 mL of PBS in thermostat shock sink at 37 °C, 50 rpm for different days (i.e., 1, 3, 5, 7, 10, 15, 20, 25 and 30 days). All experiments were performed in triplicate and the degradation results were presented as weight loss % in the following sections. In this study, the degradation percentage versus the time were applied as the desired indicator for drug release control [31,32], with more detailed information regarding the proposed degradation mechanisms of our material to be investigated and discussed in the near future.

### 2.5. Evaluation of Compression Testing Using a Universal Testing Machine (BOSE, USA) 

The test conditions of compression performances were as follows: the shape of the sample was a cylinder of diameter = 5 mm and height = 10 mm. The hydrogels were swollen in 1× PBS before the measurement, until the water content reached equilibrium. The compressive modulus with the compressive strain ratio at 70% was chosen as the compressive strength of the sample. The compression rate was 0.1 mm/s and the compressive modulus (E) was calculated as the slope of the linear portion of the stress–strain curve.

### 2.6. Biocompatibility Investigation

#### 2.6.1. Cell Cultures

MG-63 human osteoblast-like cells (ATCC CRL-1427, Rockville, MD, USA) were cultured in the culture media listed in Table 2 at 37 °C in a humidified 5% CO_2_ atmosphere for 2 days.

After thawing, they were routinely split 1:10 every 2–3 days and used at the fourth passage. MG-63 cells were detached using 0.25% trypsin in 1 mM EDTA (Sigma-Aldrich, St. Louis, MO, USA) and plated in triplicate on a 24-well microplate at a density of 5 × 10^4^ cells/well filled with a 75% (*v*/*v*) ethanol sterilized **CHTP** specimen at the density of 0.1 g/well (all of the solutions under the test were firstly sterilized using a 0.2 µm pore size membrane filter). Cells were seeded on a microplate alone for blank control. Cell growth for each condition was assessed using MTT assay after two days.

#### 2.6.2. MTT Viability Assay

After culturing MG-63 on the examined conditions for 2 days, the medium was removed; 0.2 mL of MTT (3-dimethylthiazol-2,5-diphenyltetrazolium bromide; Aldrich 135038, Sigma-Aldrich, St. Louis, MO, USA) solution (5 mg/mL in DMEM without phenol red) and 1.8 mL DMEM were added to all wells in the microplate; the multi-well plates were incubated at 37 °C for a further 4 h. After discarding the supernatants, the dark blue formazan crystals were dissolved by adding 2 mL of solvent (Dimethyl sulfoxide, DMSO) and quantified by the ELISA reader (Tecan, Sunrise remote F039300, Männedorf, Switzerland) at 570 nm. Relative absorbance results were expressed as cell viability from each condition over the control cultures (i.e., cells seeded on the microplate alone).

### 2.7. Drug Loading and Drug Release

The **CHTP** samples were loaded with the drugs by soaking in a 1× PBS solution (pH = 7.4) with the dissolved drug concentration of 1 mg/mL in a thermostat shock sink until equilibrium was attained at 37 °C under 50 rpm. The amount of the drug within the hydrogel, W_0_ (mg), could be calculated as follows:

(4) $$W_{0}=1\times V_{0}$$

where V_0_ (mL) represents the drug solution volume (at drug concentration of 1 mg/mL) adsorbed by each **CHTP** sample at equilibrium.

The drug release was carried out in sink conditions by soaking each drug-loaded hydrogel in 5 mL of PBS, at 37 °C and 50 rpm in a closed vessel, under stirring (180 rpm). At pre-determined time intervals such as day 1, 2, 3, 4, 5, 6 and 7, aliquots of 0.1 mL of the supernatant were collected and replaced by the same volume of fresh PBS solution. At the end of the experiment, a total of 0.7 mL of the release solution had been substituted by the fresh medium. The drug concentration values were quantified using a spectrophotometer UV–VIS (GeneQuant 1300, GE Healthcare Life Science, Pittsburgh, PA, USA) at wavelengths of 360 nm. All measurements were taken at least in triplicate. The amount of the released drug, W_t_, could be measured using the formula:

(5) $$W_{t}=M_{t}\times V_{t}$$

where M_t_ and V_t_ represent the released drug concentration and the collected drug solution volume at each time interval, respectively. The released efficiency of the drug release test was then defined as:

(6) $$\mathrm{released}\;\mathrm{efficiency}\;\left(\%\right)=W_{t}/W_{0}\times 100\%$$

### 2.8. Statistical Analysis

Statistical analysis was conducted at least in triplicate, and the results are reported as mean ± standard deviation (SD). A one-way ANOVA analysis was used to evaluate the differences between various experimental and control groups. The *p* value of <0.05 was considered statistically significant.

## 3. Results and Discussions

This pilot study provided a series of in vitro analyses of a composite material capable of being used as a drug delivery system composed of oxidized hyaluronic acid (HA)/type I collagen hydrogel integrated with β-tricalcium phosphate (β-TCP) and crosslinked with oligomeric proanthocyanidins (OPCs) as natural cross-linking agents. The corresponding results regarding the crosslinking property, the mechanical strength, swelling ratio and in vitro degradation rate of the composite material were investigated and discussed; while its biocompatibility was also evaluated using the MG-63 cell line and its application as a carrier matrix for delivery of tetracycline hydrochloride (TH) was also investigated and discussed in this section.

### 3.1. CH Hydrogel

#### 3.1.1. Characterization of Oxidized Hyaluronic Acid/Collagen Hydrogels (CH)

Mixing oxidized hyaluronic acid with the collagen solution at different concentrations was carried out to find the optimized ratio to form porous and interconnected structures inside the hydrogel for carriers of bioceramics and drugs which may result in better control of its mechanical properties, resistance to degradation and biotic infections.

**Figure 1:**
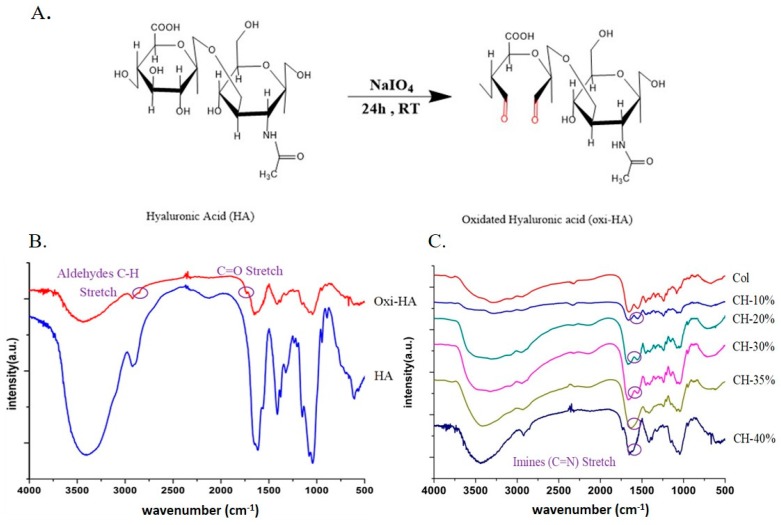
(**A**) Chemical schematic of hyaluronic acid oxidation oxidized by sodium periodate; the newly formed aldehyde group is expressed in red color [37]; (**B**) FTIR Spectra of hyaluronic acid (red) and oxidized hyaluronic acid (blue); and (**C**) collagen (Col) and oxi-HA/collagen (CH) at different weight ratios (*w*/*w*).

To make an oxidized HA, sodium periodate, used widely as an oxidizing agent, was used to create the functional group of hyaluronic acid available to crosslink with collagen to form an injectable hydrogel in this study [33], which can further be used as the carrier of β-TCP and drugs. Our first step to prepare our composite material was to have the dialdehyde groups introduced on HA (Figure 1A) by reacting with NaIO_4_, via opening the glucuronic acid ring and oxidizing the proximal OH groups. After the reaction process, the FTIR spectrum (Figure 1B) was used to confirm the formation of dialdehyde groups (Figure 1A) with the observation that there were two newly formed peaks at 1733 cm^−1^ and 2810 cm^−1^ which associates with the C=O and C–H stretch of oxi-HA, respectively [33,34].

The successful oxidization of HA was further confirmed by the absorption peak (Figure 1C) at 1630 cm^−1^ which associates with the C=N stretch, which resulted from interacting its aldehyde group (C=O) with the free –NH_2_ group of collagen to make CH polymers. In this case, collagen without crosslinking shows the broader absorption peak around 3300 cm^−1^ from the FTIR spectrum associated with its amide A group due to the NH stretch in resonance with an amide II overtone [35,36].

#### 3.1.2. SEM Morphology of CH Hydrogel

**Figure 2:**
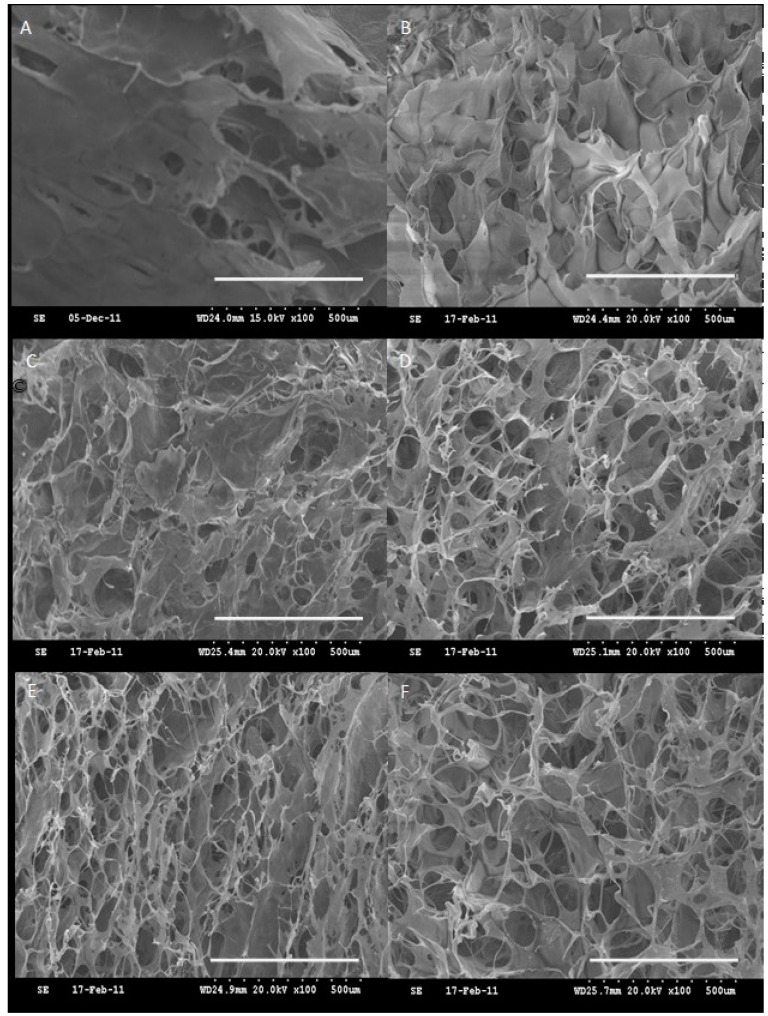
SEM morphology (scale bar = 500 μm) of (**A**) lyophilized collagen and oxi-HA/collagen hydrogel (CH) at different concentrations (*w*/*w*) after brief fixation and serial dehydration; with the connective pores clearly shown in (**B**) CH-10%; (**C**) CH-20%; (**D**) CH-30%; (**E**) CH-35% and (**F**) CH-40%.

The oxi-HA/collagen (CH) hydrogels made from mixing HA with the collagen solution at different weight percentages were pre-formed and freeze-dried to observe the cross-section after liquid nitrogen immersion. Figure 2 shows the SEM morphology of CH hydrogels, with the clear trend observed that collagen and oxi-HA were able to crosslink with each other and form porous structures inside the hydrogel. Interconnecting pores were obviously observed in the hydrogel matrix with more consistent pore sizes ranging from 50 to 250 μm when the weight percentage of oxi-HA was higher than 30, probably due to the saturation of the crosslinking within the hydrogel, which is evaluated and discussed in the next section. 

The mean pore size has been shown to impact tissue regeneration after implantation. Within the scaffold porosity controlled in our hydrogels at a diameter ranging from 50 to 250 μm, it was good for the regeneration of bone, osteoid ingrowth and for the regeneration of adult mammalian skin [38]. Thus, the interconnecting pores and hydrogel microarchitectures are suitable for cell survival in a 3D environment, and most importantly, could be used for the transportation of bioceramics such as β-TCP and for the drugs mentioned in the following sections. In this study, SEM pictures were specifically applied to investigate the porous structures of CH material in morphology, which were not applicable to CHT or CHTP in similar studies since the textures of the porous space were obscured by the added TCP (i.e., Figure S1 in supporting information).

#### 3.1.3. Cross-Linking Index

**Figure 3:**
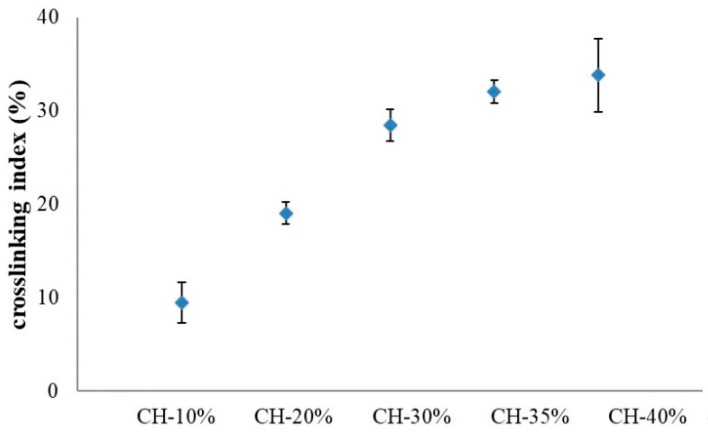
Crosslinking index of CH hydrogels made from oxidized HA/collagen at different concentrations (*w*/*w*). Error bars represent standard deviations of triplicate measurements.

The degree of cross-linking of the CH polymer was calculated as the cross-linking index, with a higher index value representing less amount of residual and free –NH_2_ groups observed in the mixture of oxi-HA and collagen. As shown in Figure 3, the crosslinking index increased with the amount of oxidized HA within the CH polymer, and reached a steady state when the weight percentage of oxi-HA was higher than 30. As the result, CH-35% was chosen as the optimized ratio of oxidized HA/collagen for the carrier of β-TCP in our later study, which also implies that the free-NH_2_ group in collagen under the test might have been consumed completely at this ratio and resulted in a more consistent porous size shown in Figure 2 (morphology of SEM pictures of Figure 2D–F). 

In our composite material design, our next step is to synergize the advantageous properties of both ceramics and natural polymers by incorporating β-tricalcium phosphate (β-TCP) into our CH-35% hydrogel to make a CHT polymer, with its swelling, mechanical and degradation test results shown and discussed below.

### 3.2. CHT Composite Material

#### 3.2.1. Swelling and Mechanical Test 

**Figure 4:**
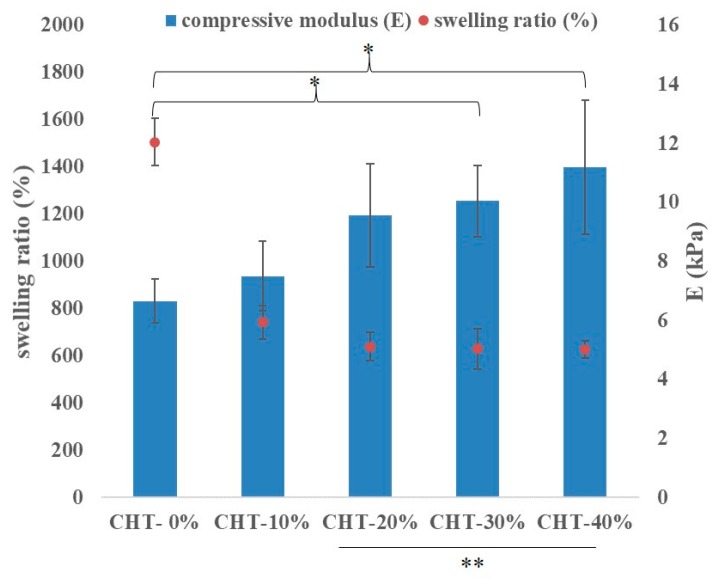
Swelling ratio (%) (data collected at soaking time of 120 min) (left y-axis) and compression modulus (E) (right y-axis) of CHT with different β-TCP content. Error bars represent standard deviations of triplicate measurements. * indicates a significant difference (*p* < 0.05). ** represents no significant difference (*p* > 0.05).

As shown in Figure 4, changes in the amount of β-tricalcium phosphate (β-TCP) added altered not only the compositions and molecular weight of composite hydrogels but also their swelling percentages. The results show that the incorporation of the β-TCP into the CH-35% composite leads to a reduction in swelling percentage in all samples. The control sample, CH-35%—oxidized HA mixed with collagen at a weight percentage of 35% (*w*/*w*) without any of the β-TCP added—which is also called CHT-0%, had a significantly higher swelling percentage than that of any other CHT-based hydrogel with β-TCP added at different concentrations. This reduction in swelling percentage may be due to the following reasons: (i) The empty voids within the CH polymeric scaffold were occupied by β-TCP. (ii) The interactive forces between oxi-HA, collagen and β-TCP doped reduced the distance between the polymer chains making up the hydrogel. This enhanced interactions may further induce greater mechanical modulus, for example, the compression modulus, with the higher modulus represents stronger resistance to the compressive force experienced from the surrounding tissues when the material is placed in vivo [39,40]. This trend was observed from the compression testing data shown in Figure 4 below, with the compression modulus increasing with the amount of β-TCP added. The effects of the β-TCP added to these properties of CHT material (e.g., swelling ratio and compressive modulus) reached a plateau when the TCP content was beyond 20% in weight, probably due to the fact that the void space in CHT was becoming saturated by the TCP contents. 

The trend observed in Figure 4 is not a surprising result since when a hydrogel-based composite begins to swell, it continues to absorb the solvent until the equilibrium between the osmotic pressure from the swelling and the elasticity of the polymer network is reached [41]. In this case, the swelling properties in hydrogels might be directly related to the degree and type of crosslinking presented [42], which might be able to alter the mechanical properties of hydrogel-based composites as well, with an increase in the percentage swelling negatively affecting the mechanical properties of hydrogels as shown in Figure 4 due to the changes in the distances between polymer chains, which might further influence cell migration and the releasing rate of β-TCP powders and drugs [39]. In summary, the controlled β-TCP content plays a critical role for tissue engineering especially when many of the physical properties of composite scaffolds are involved.

Although the β-TCP added can increase the compression modulus of the CHT composite material, the degradation data shown in the next section revealed that a higher content of β-TCP leads to a higher weight loss of the CHT composite from which an additional crosslinking agent is required to retain the β-TCP in the hydrogel for a longer period of time or for better control in its degradation rate.

#### 3.2.2. Degradation Test

**Figure 5:**
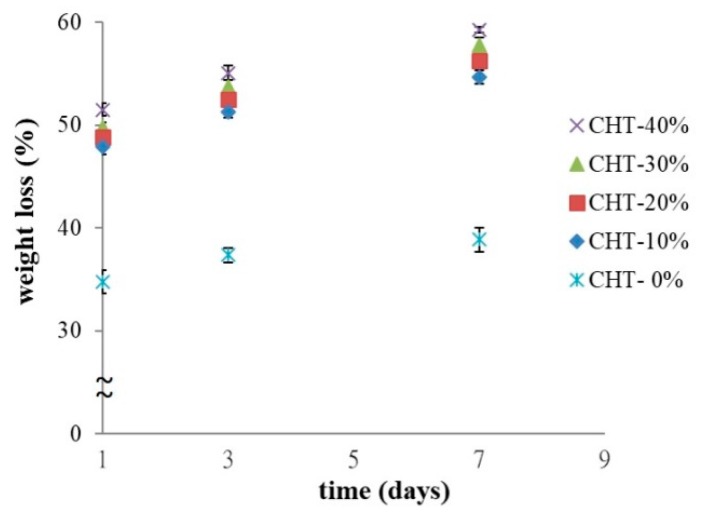
Effect of the amount of β-TCP added on the degradation percentage of CHT composite materials. Error bars represent standard deviations of triplicate measurements.

The effects of the β-TCP content in a CHT polymer composite on degradation are shown in Figure 5. The oxi-HA/collagen content ratio is fixed at 35% (*w*/*w* %) in all cases. As shown, a higher content of β-TCP in the weight percentage of CHT leads to more weight loss (i.e., 50% of total weight loss in 7 days in PBS at 37 °C); this is probably due to two reasons when β-TCP was involved in degradation. The first is that the β-TCP was physically entrapped within the composite polymer scaffolds with weaker interactive forces involved to retain the β-TCP content which leads to the increased degradation rate. The second reason is that accompanying the degradation of the entire polymer scaffold was the constraint on keeping the β-TCP content in the composite from getting weaker which resulted in the speeding up of weight loss. As the result, additional crosslinking is required to defer the degradation of CH polymer scaffolds and to provide additional interactive forces to retain the β-TCP within the CHT composite material.

Considering the options of potential crosslinkers, a natural crosslinking agent such as proanthocyanidins (PA) has been successfully used in the pretreatment of biological tissues to improve their mechanical properties, and it overcomes some of the drawbacks that are typically encountered with synthetic cross-linking agents (e.g., formaldehyde, glutaraldehyde (GD), epoxy compounds, and carbodiimide) such as the high cytotoxicity which weakens the hydrogel’s long-term biocompatibility [43,44].

Although it is unknown what minimum concentration of proanthocyanidins is needed for the protection of collagen from degradation, recent studies have used a 5% PA concentration to show the collagen crosslinking capability [43,45], with three possible mechanisms proposed to protect collagen from degradation. For example, OPCs were reported to cause irreversible conformational changes in proteases, modulate protease production and activation through host immune systems and to increase the crosslinking density of the collagen network, thereby enhancing the matrix resistance against enzymatic degradation over time [46]. Based on these reasons, 5% (*w*/*w*) of the OPCs were applied to a CHT-30% to make the CHTP in our study.

### 3.3. CHTP Composite Material

CHT-30% was made by mixing CH-35% and β-TCP at a weight ratio of CH:β-TCP = 0.7:0.3 (*w*/*w*), which is fixed in all cases to present the overall properties of a CHT composite material for the OPC studies discussed in this section.

#### 3.3.1. Degradation Test

**Figure 6:**
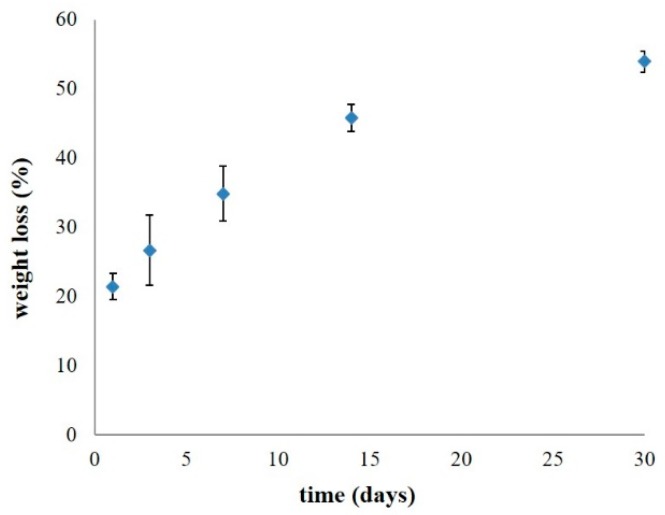
Degradation test results of CHTP. Error bars represent standard deviations of triplicate measurements.

As shown in Figure 6, the weight loss (%) was less than 50% within 20 days in 1× PBS at 37 °C, which shows a much slower degradation rate when compared to any of the CHT composites with β-TCP added (i.e., Figure 5), probably due to a higher crosslink density caused by OPCs in our CHTP design, which requires more cleavage of these crosslinks to completely degrade the hydrogel.

From these investigated data, CHTP might be an optimized design of composite material for further study as a drug delivery system, with its corresponding test results on swelling properties, mechanical test results, biocompatibility and drug delivery properties shown and discussed in the following sections.

#### 3.3.2. Characterizations of CHTP Composite Material 

**Figure 7:**
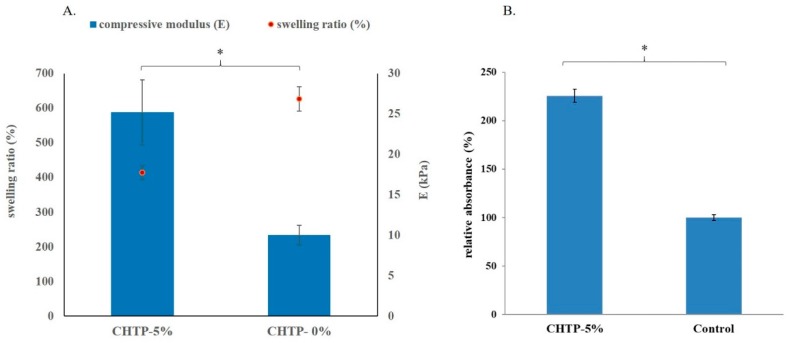
(**A**) Swelling ratio (%) (data collected at soaking time of 120 min) (left y-axis) and compression modulus (E) (right y-axis) of CHT with and without OPCs added. CHTP-5% represents that 5% OPCs were applied to a CHT composite with 30% (*w*/*w*) of β-TCP added (CHT-30% or CHTP-0%); (**B**) Cell viability evaluated by MTT assay for CHTP composite material. Cell growth is expressed as the relative absorbance obtained over the control condition. Error bars represent standard deviations of triplicate measurements. * indicates significant difference (*p* < 0.05).

In this section, CHT-30% was applied with 5% OPCs as cross-linker for the rest of the tests, which could be called CHTP or CHTP-5%; when CHT-30% was used alone without the OPCs applied, it could be called CHTP-0% as a control sample. Here, as shown in Figure 7A, the effect of 5% of OPCs added resulted in stronger interactions between polymer chains as well as a reduced swelling percentage compared to the control sample, CHT-30% (or CHTP-0%). This reduction in swelling percentage thus induces greater compression strength in the overall hydrogel-based composites, with the compression modulus increasing when OPCs were applied. This enhanced mechanical property was also beneficial to the alveolar bone reconstruction during tissue engineering [8].

The MTT assay was also applied to determine the viability of cells grown on the CHTP composite material. Results showed that there were even better cell viability (%) values for cell growth on the CHTP compared to the control sample (Figure 7B), which suggests that this material design can be biocompatible and meets our needs.

The CHTP was specifically designed as the composite scaffold to mimic the natural composition of bone. A composite of ceramic and biopolymer ensures better cell migration; mechanical stability and osteoconductive properties. Such properties are critical to the bone healing process due to better cellular attachment, proliferation and differentiation [47,48,49], represented by higher osteoblast viability or estimated by a higher number of alive cells counted. More specifically, the addition of the OPCs was reported to stimulate bone formation through its positive action on cellular differentiation or protection as an antioxidant against oxidative stress [50,51,52,53]. Due to these combined effects, it is no surprise that the CHTP provides better cell viability than the control group observed.

#### 3.3.3. Drug Releasing Profile

**Figure 8:**
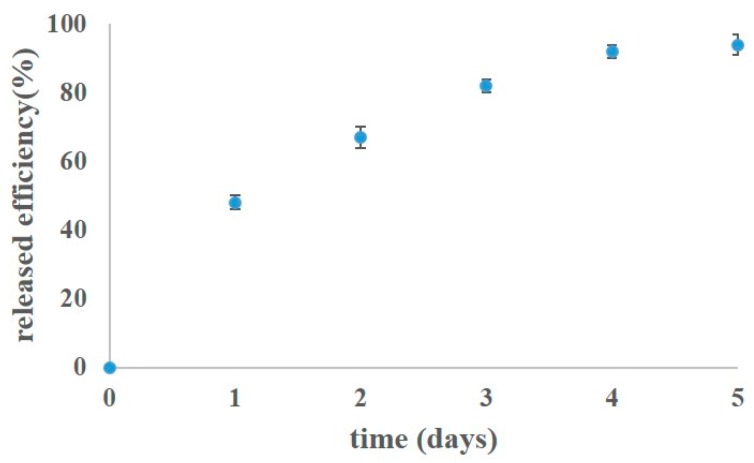
Drug release profile for tetracycline-loaded CHTP composite material. Error bars represent standard deviations of triplicate measurements.

Our final objective was to fabricate a novel antibacterial GBR material to prevent infection. As a result, the CHTP composite material was investigated regarding its capability to deliver drugs using tetracycline in this study. Tetracycline was chosen because of its antimicrobial resistance properties, its potential to minimize the risk of post-surgery infection and its clinical relevance for use in the treatment of osteomyelitis [54,55,56]. The drug release profile for the tetracycline-loaded CHTP composite is illustrated in Figure 8. The first region (burst effect: 50% release after 24 h) might be a result of the release of drug molecules at the surface and in close proximity to the surface of the CHTP composite. The second region (sustained release: 93% release after 5 days) might be due to the diffusion and swelling mechanism with the expansion of pores allowing the drug molecules to escape freely out of the CHTP, with 230 mg of 100% drug released per gram of CHTP under the test. 

The initial burst release has the advantage of sterilizing the infected area from the first day, which is followed by a sustained release to keep an aseptic surface of the scaffold [39]. The burst effect and controlled drug release duration were probably dependent on β-TCP loading, due to the abruption of β-TCP particles from the cross-linked structures within the CHTP, allowing the drug molecules to diffuse out of the hydrogel-based composite much more easily.

## 4. Conclusions

A collagen-based hydrogel was prepared and designed using oxidized HA and OPCs as the natural cross-linkers for the carrier of β-TCP and antibacterial drugs. The effects of varying the formulation ratios of the integrated composite materials were investigated in terms of the physical, mechanical and chemical properties of the resulting hydrogel-based composites.

Collectively, these results indicate that this composite material had a high swelling ratio of 420% and mechanical strength of 25 kPa while remaining at more than 60% of the weight after 30 days of an in vitro degradation test with good biocompatibility in promoting the proliferation of MG-63 cells. Additionally, drug dissolution studies showed the burst effect of 50% release after 24 h and 93% release after 5 days, with 230 mg of tetracycline released in total per gram of **CHTP** under the test. With the introduction of an antimicrobial drug (tetracycline), this composite material design would then be a potential candidate for an antibacterial GBR material in the treatment of advanced chronic periodontitis.

## Figures and Tables

**Table 1:** Polymeric composites made in this study.

Polymer	Components	Abbreviation
A	Oxidized hyaluronic acid	(oxi-HA)
B	A + Collagen	CH
C	B + β-tricalcium phosphate (β-TCP)	CHT
D	B + β-TCP + OPCs	CHTP

**Table 2:** Components of MG-63 cell culture medium.

Name	Concentrations in g/L or mL/L
Minimum essential medium (MEM)	9.3918 g
Non-essential amino acid (NEAA)	10 mL
Sodium pyruvate (SP)	10 mL
L-glutamine (L-G)	10 mL
Prostate-specific antigen (PSA)	10 mL
Sodium bicarbonate	1.5 g
Heat-inactivated fetal bovine serum	100 mL

## Supplementary Materials

The following are available online at http://www.mdpi.com/1999-4923/10/2/37/s1, Figure S1: SEM morphology of CHT and CHTP.
