# Supplementary material for: Integrated Oxidized-Hyaluronic Acid/Collagen Hydrogel with β-TCP Using Proanthocyanidins as a Crosslinker for Drug Delivery
Source: Pharmaceutics. 2018 Mar 21;10(2):37. doi: 10.3390/pharmaceutics10020037 (PMC6030783; doi:10.3390/pharmaceutics10020037)
Supplement: Supplementary file 1 [file pharmaceutics-10-00037-s001.pdf]

Supplementary Materials: Integrated Oxidized-Hyaluronic Acid/Collagen Hydrogel with β-TCP Using Proanthocyanidins as a Crosslinker for Drug Delivery

Yang Wei, Yu-Han Chang, Chung-Jui Liua and Ren-Jei Chung

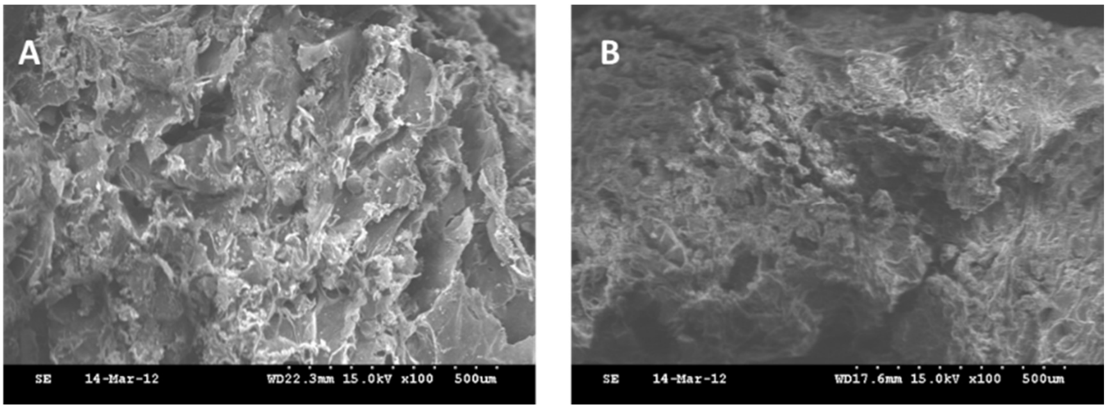

Figure S1. SEM morphology (scale bar=500μm) of (**A**) CHT and (**B**) CHTP.
